# Low Total Testosterone Levels in Men with Newly Diagnosed Early-Onset Type 2 Diabetes: A Cross-Sectional Study in China

**DOI:** 10.1155/2023/2082940

**Published:** 2023-05-02

**Authors:** Yun Hu, Lu Yuan, Yu-jie Bao, Ying Wang, Ting-ting Cai, Jian-hua Ma, Bo Ding

**Affiliations:** ^1^Department of Endocrinology, The Affiliated Wuxi People's Hospital of Nanjing Medical University, Wuxi Medical Center, Nanjing Medical University, Wuxi, China; ^2^Department of Endocrinology, Nanjing First Hospital, Nanjing Medical University, Jiangsu, Nanjing, China

## Abstract

**Objective:**

There is a bidirectional interaction between circulating testosterone and blood glucose levels. We aim to investigate the testosterone levels in men with early-onset type 2 diabetes (T2DM).

**Methods:**

A total of 153 drug naive men with T2DM were enrolled in the study. Early- (*n* = 63) and late-onset (*n* = 90) T2DM was classified according to age 40 years old. Clinical characteristics and plasma for biochemical criterions were collected. Gonadal hormones were measured using chemiluminescent immunometric assay. The concentrations of 3*β*- and 17*β*-HSD were determined using ELISA.

**Results:**

Compared with men with late-onset T2DM, those with early-onset T2DM had lower serum total testosterone (TT), sex hormone-binding globulin (SHBG), and FSH, but higher dehydroepiandrosterone sulfate (DHEA-S) level (*p* < 0.05). The mediating effect analysis showed that the decreased TT levels in patients with early-onset T2DM were associated with the higher HbA1c, BMI, and triglyceride in these patients (both *p* < 0.05). The early-onset of T2DM directly correlated with increased DHEA-S (both *p* < 0.01). The 3*β*-HSD concentration in the early-onset T2DM group was lower than that in the late-onset T2DM group (11.07 ± 3.05 vs. 12.40 ± 2.72 pg/mL, *p* = 0.048) and was positively correlated with fasting C-peptide, while negatively correlated with HbA1c and fasting glucagon (*p* all < 0.05).

**Conclusions:**

Patients with early-onset T2DM showed inhibition of conversion from DHEA to testosterone, which may attribute to the low level of 3*β*-HSD and high blood glucose in these patients.

## 1. Introduction

Prevalence of type 2 diabetes mellitus (T2DM) is rapidly increasing worldwide and observed increasingly among younger adults in China [1]. Early-onset T2DM was defined as diagnosis before 40 years of age [2]. According to the Joint Asia Diabetes Evaluation (JADE) programme, 18% of Asian adults with T2DM were early onset [3]. In China, the prevalence of T2DM among 18-29 and 30-39 years old was 4.5% and 6.6%, respectively, and the prevalence of prediabetes aged <40 was as high as 40-50% [4].

Compared with late-onset T2DM, patients with early-onset T2DM had more severe metabolic disorders and diabetic complications [5]. Early-onset T2DM is also associated with more unfavorable cardiovascular disease risk factors as well as greater morbidity and mortality when compared with T1DM [6]. A cross-sectional study using data from China National HbA1c Surveillance System showed that Chinese patients with early-onset T2DM were at increased risk of nonfatal cardiovascular disease [7].

Testosterone is a hormone that plays a key role in carbohydrate, fat, and protein metabolism, and it has been known for some time that testosterone has a major influence on body fat composition and muscle mass in males, both of which are closely related to insulin resistance [8]. Previous study showed that the prevalence of testosterone deficiency was significantly higher in men with type 2 diabetes than in the normal population (51% vs. 30%) [9], and long-term testosterone therapy prevented prediabetes progression to T2DM in men with hypogonadism [10].

In normal men, testosterone levels decreased about 0.3–2.0% per year naturally [11]. However, Li et al. found that patients with early-onset T2DM showed much higher prevalence of hypogonadism than those with late-onset T2DM (48.0% vs. 26.7%) [12]. Unfortunately, the study was performed in patients with the age of 18~80 years, and the mean age of late-onset group was 64.56 ± 10.74 years. It has been demonstrated that testosterone begins to drop after the age of 30~40, but more quickly after 65 years [13, 14]. As a result, Li et al. did not find a difference in testosterone levels between the patients with early- and late-onset T2DM [12]. Therefore, we performed the present study to compare the levels of gonadal hormones between men with early-onset T2DM and those with late-onset T2DM before 65 years old and attempt to investigate the role of metabolic indicators and hydroxysteroid dehydrogenase (HSD) levels in the relationship between the age of T2DM onset and testosterone levels.

## 2. Materials and Methods

### 2.1. Subjects

The present study is a subsequent research of our previous studies (clinicaltrials.gov, NCT03982238) [15, 16]. We consecutively included the patients who were admitted to Nanjing First Hospital from March 2021 to May 2022 because of high blood glucose with or without diabetes symptoms and met the inclusion criteria. The inclusion criteria were as follows: newly diagnosed male T2DM patients who met the World Health Organization (WHO) 1999 diagnostic criteria, aged 18-65 years, and had not been treated with any hypoglycemic drugs. Exclusion criteria were as follows: (1) already on treatment with lipid-lowering or antihypertensive medications, (2) with serum alanine aminotransferase (ALT) level more than 2.5 times the upper normal range (100 U/L) or creatinine level more than 1.3 times the upper normal range (105*μ*mol/L), (3) a history of systemic corticosteroid use in the past 3 months, and (4) any infection or acute diabetic complications such as ketoacidosis or hyperosmolar state (coma). The classification of type 2 diabetes was judged by at least two endocrinologists, and type 1 diabetes was excluded according to the “Standards of Medical Care for Type 2 Diabetes in China” (2017) [17]: usually under 30 years old; obvious symptoms of polydipsia, polyuria, polyphagia, and emaciation begin with ketosis or ketoacidosis and nonobese; fasting or postprandial serum C-peptide concentration significantly decreased; positive autoimmune markers include islet cell autoantibodies (ICA-Ab), autoantibodies to glutamic acid decarboxylase (GAD-Ab), tyrosine phosphatases IA-2, and zinc transporter 8 (ZnT8). We performed ICA-Ab and GAD-Ab in the present study at the same time as the patients were diagnosed with diabetes.

### 2.2. Anthropometric and Laboratory Measurements

After admission, baseline parameters including age, height, weight, waist circumference (WC), and medical history were collected. Body mass index (BMI) was calculated as weight divided by the square of height (kg/m^2^). Blood samples were collected following an overnight fast for the assessment of biochemical indicators. Fasting blood glucose (FBG), creatinine (Cr), total cholesterol (TC), triglyceride (TG), high-density lipoprotein (HDL), low-density lipoprotein (LDL), apolipoprotein A-1 (ApoA-1), and apolipoprotein B (ApoB) were measured by an autoanalyzer (Modular E170; Roche, Mannheim, Germany). Glycated hemoglobin (HbA1c) was determined using high-performance liquid chromatography assay (Bio-Rad Laboratories, USA). Fasting C-peptide (FC-p), fasting glucagon (FGLA), postprandial C-peptide (PC-p), postprandial glucagon (PGLA), and gonadal hormones including follicle-stimulating hormone (FSH), luteinizing hormone (LH), estradiol (E), progestin (P), prolactin (PRL), testosterone (T), and dehydroepiandrosterone sulfate (DHEA-S) were measured with chemiluminescent microparticle immunoassay (Architect system, USA). GAD-Ab (reference range, <10 IU/mL) and ICA-Ab were tested using chemiluminescent immunoassay (YHLO, China). 3*β*-HSD and 17*β*-HSD were determined by enzyme-linked immunosorbent assay following the manufacturer's recommendations (Jiangsu Meimian Industrial, China). Both the intra- and interassay coefficients of variation were less than 10%. Free testosterone (FT) and bioactive testosterone (Bio-T) were calculated through the web site http://www.issam.ch/freetesto.htm. Homeostatic model assessment of insulin resistance (HOMA-IR) index was determined with FBG and FC-P through HOMA2 calculator software (download from http://www.dtu.ox.ac.uk/homacalculator/download.php) [18].

### 2.3. Statistical Analysis

Data are presented as mean ± standard error (SE), median (interquartile range), or percentage as appropriate. Standard *t* test was used to compare normally distributed data, and the Wilcoxon test was used for asymmetrically distributed data. The categorical data were examined with chi-square test. The Pearson or Spearman correlation analysis was performed between biochemical indicators and the levels of sex hormones. Mediation analysis was conducted to explore the potential mediating effects on the association of gonadal hormone with the age of diabetes onset. All statistical analyses were performed using SPSS version 22.0 software (IBM Corp., USA). A *p* value < 0.05 was considered statistically significant.

## 3. Results

### 3.1. General Characteristics of Patients

Finally, a total of 153 men with newly diagnosed T2DM with the mean age of 44.99 ± 0.88 years were recruited in this study. The mean BMI was 25.73 ± 0.29 kg/m^2^, and the HbA1c was 9.99 ± 0.17%. According to the diagnosis age before 40 years or not, we divided patients into two groups: early-onset group (*n* = 63) and late-onset group (*n* = 90). Compared to patients with late-onset T2DM, those with early-onset T2DM had higher BMI and WC. There were no significant differences in the proportion of smoking and hypertension between the two groups (Table 1).

### 3.2. Biochemical Characteristics between Two Groups

As shown in Table 1, compared to patients with late-onset T2DM, those with early-onset T2DM had higher HbA1c and severe dysfunction of lipid metabolism (TC, TG, ApoB, and ApoB/ApoA-1). However, the level of FBG, FC-p, FGLA, PC-p, PGLA, HDL, LDL, ApoA-1, and HOMA-IR had no significant differences between the two groups.

### 3.3. Sex Hormone Concentrations between Two Groups

**Figure 1:**
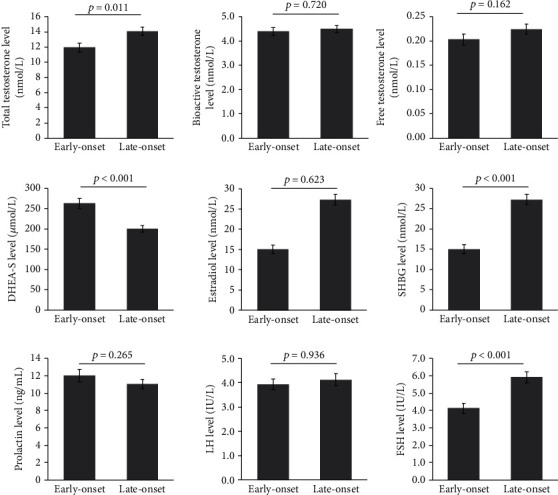
Serum sex hormone concentrations between patients with early-onset and late-onset T2DM. T2DM patients were divided into early-onset diabetes group (*n* = 63) and late-onset diabetes group (*n* = 90) according to the age of onset of diabetes at 40 years. DHEA-S: dehydroepiandrosterone sulfate; SHBG: sex hormone binding globulin; LH: luteinizing hormone; FSH: follicle-stimulating hormone.

As shown in Figure 1, patients with early-onset T2DM had lower TT (11.93 ± 0.57 vs. 14.04 ± 0.56 nmol/L, *p* = 0.011), FSH (4.13 ± 0.3 vs. 5.92 ± 0.32 IU/L, *p* < 0.001), and SHBG (15.03 ± 1.07 vs. 27.29 ± 1.26 nmol/L, *p* all < 0.05), but higher level of DHEA-S (263.08 ± 12.66 vs. 200.17 ± 8.00 *μ*mol/L, *p* < 0.001). Therefore, the ratio of T/DHEA-S was significantly decreased in patients with early-onset of T2DM (0.02 ± 0.001 vs. 0.03 ± 0.003 nmol/*μ*mol, *p* = 0.001) compared with the late-onset group. There were no significant differences in E, LH, RPL, Bio-T, and FT concentration between two groups (*p* all > 0.05).

### 3.4. Mediating Effects between the Sex Hormone Concentrations and the Age of Diabetes Onset

**Figure 2:**
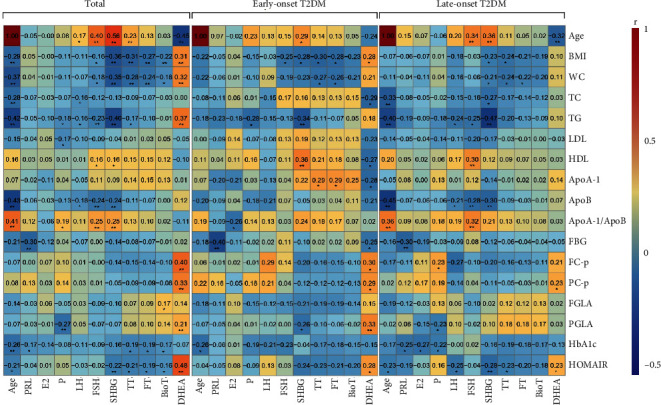
The correlation analysis between sex hormone concentrations and metabolic indexes. The Pearson/Spearman correlation analysis was performed between biochemical indicators and the levels of sex hormones in 153 patients with T2DM (total) and was also performed in the early-onset T2DM (*n* = 63) and late-onset T2DM (*n* = 90) groups separately. Heatmap showing the correlation coefficient (*r*) levels. ^∗∗^*p* < 0.01 and ^∗^*p* < 0.05. FGLA: fasting glucagon; PGLA: postprandial glucagon; HOMA-IR: homeostatic model assessment of insulin resistance; FC-p: fasting C-peptide; PC-p: postprandial C-peptide; ApoA-1: apolipoprotein A-1; ApoB: apolipoprotein B; HDL: high-density lipoprotein; LDL: low-density lipoprotein; TG: triglyceride; TC: total cholesterol; FBG: fasting blood glucose; HbA1c: glycated hemoglobin; BMI: body mass index; WC: waist circumference; DHEA: dehydroepiandrosterone sulfate; SHBG: sex hormone binding globulin; LH: luteinizing hormone; FSH: follicle-stimulating hormone; E2: estradiol; P: progestin; PRL: prolactin; TT: total testosterone; FT: free testosterone; Bio-T: bioactive testosterone.

TT was positively correlated with age and was also correlated with BMI, WC, HbA1c, TG, and HOMA-IR (*p* all < 0.05, Figure 2) according to the correlation analysis. DHEA-S was negatively correlated with age and significantly correlated with BMI, WC, TG, fasting and 2 h C-peptide, HOMA-IR, and 2 h glucagon (*p* all < 0.05). In both groups, TT remains negatively correlated with BMI and WC, and DHEA-S remains positively correlated with 2 h C-peptide and HOMA-IR. Moreover, TT was positively correlated to ApoA-1 in patients with early-onset T2DM and negatively correlated with HOMA-IR in late-onset T2DM (*p* all < 0.05). DHEA-S was positively correlated with BMI, FC-P, and PGLA and negatively correlated with TC, HDL, ApoA-1, and FBG in patients with early-onset T2DM only. After age stratification, the correlation between TT and age, as well as DHEA-S and TG, was no longer significant (*p* all > 0.05, Figure 2).

**Figure 3:**
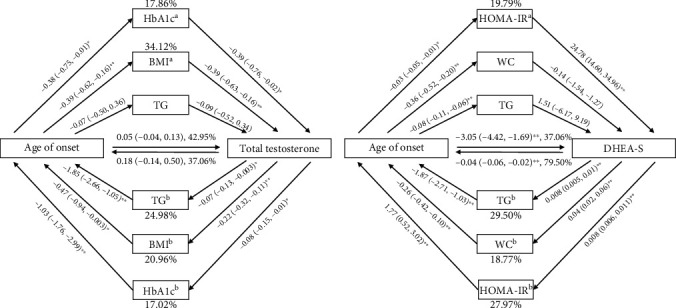
Mediating effect analysis between the sex hormone concentrations and the age of diabetes onset. (a) The mediating effects of triglyceride (TG), body mass index (BMI), and HbA1c between age of diabetes onset and total testosterone. (b) The mediating effects of triglyceride (TG), waist circumference (WC), and HbA1c between age of diabetes onset and dehydroepiandrosterone sulfate (DHEA-S). ^∗∗^*p* < 0.01 and ^∗^*p* < 0.05.

We performed a mediating analysis between the age of T2DM onset and the levels of TT and DHEA-S using the related factors. The results showed that the total effect of the age of T2DM onset on the TT levels was 0.11 (0.03 and 0.18) and the total effect of the TT levels on the age of T2DM onset was 0.49 (0.15 and 0.82), both *p* = 0.005. As shown in Figure 3(a), without significant direct effect, the age of T2DM onset had significant indirect effect on TT levels through HbA1c and BMI (both *p* < 0.05), and TT levels also had indirect effect on the age through HbA1c, BMI, and TG (*p* all < 0.05). Among these factors, BMI accounts for 34.12% of the total effect of age on TT, and TG accounts for 24.98% of the total effect of TT on the age of diabetes onset. The total effect of age on DHEA-S was -3.90 (-5.20 and -2.61), and that of DHEA-S on age was -0.05 (-0.06 and -0.02), both *p* < 0.001. There were direct interactions between DHEA-S and age of diabetes onset (both *p* < 0.01, Figure 3(b)). Moreover, HOMA-IR had mediating effects between DHEA-S and age (both *p* < 0.05, Figure 3(b)), while TG and WC only mediated the effect of DHEA-S on the age.

### 3.5. The 3*β*-HSD and 17*β*-HSD Concentrations in Early-Onset T2DM

We further measured 3*β*- and 17*β*-HSD in 76 patients to explore the potential reason of the reduction of T/DHEA-S ratio, and the 3*β*-HSD concentration in the early-onset T2DM group (*n* = 33) was lower than that in the late-onset T2DM group (*n* = 43, 11.07 ± 3.05 vs. 12.40 ± 2.72 pg/mL, *p* = 0.048). However, there was no significant difference of 17*β*-HSD concentration between the two groups (28.63 ± 2.00 vs. 25.88 ± 1.63 pg/mL, *p* = 0.287). Moreover, linear regression analysis showed that the 3*β*-HSD level was correlated with fasting C-peptide (standardized *β* = 0.576, *p* = 0.024), HbA1c (standardized *β* = −0.317, *p* = 0.034), and fasting glucagon (standardized *β* = −0.357, *p* = 0.038), while BMI and WC, blood lipids, and HOMA-IR were excluded.

## 4. Discussion

The present study describes the characteristics of circulating sex hormone levels in men with early-onset T2DM. Our results showed significant decreased serum TT, SHBG, and FSH levels and increased DHEA-S level in men with early-onset T2DM compared with men with late-onset T2DM, which have not been reported previously as we are aware of.

Previous cross-sectional and longitudinal data indicated that testosterone falls progressively with age [19]. To our surprise, TT levels were lower in men with early-onset T2DM. The result explained the higher prevalence of hypogonadism in men with early-onset T2DM than those with late-onset T2DM in the previous study [12]. Another study in patients with T2DM also showed higher prevalence of hypogonadism in men within the range of 31 and 40 years old (29%) than in men from 41 to 50 years old (12%) [20]. Therefore, our results indicate that it is necessary to measure testosterone levels in men with early-onset T2DM.

DHEA, a steroid hormone mainly synthesized by the adrenal cortex, is an important intermediate in the synthesis of testosterone and exists in the main form of DHEA-S [21]. Both DHEA-S and DHEA plasma levels declined with age in healthy men [22]. In the present study, DHEA-S was also significantly declined with age in patients with T2DM. DHEA is oxidized by 3*β*-HSD on the 3*β*-hydroxy group to generate Adione, later then catalyzed by 17*β*-HSD and results in a reduction of its C17 keto group to a *β*-hydroxyl group, and finally generates testosterone [21]. Due to the low TT and high DHEA-S in men with early-onset T2DM, it is reasonable to suspect that there is a disorder in the conversion from DHEA to testosterone in these patients, and the low level of 3*β*-HSD in men with early-onset T2DM in this study confirmed our hypothesis.

The present study found that SHBG and FSH levels were lower in the early-onset group than those in the late-onset group. Previous study also showed an age-related increase of SHBG (about 1.2% per year) with an increase of fat mass and insulin levels [22], and Li et al. found significant reduced FSH levels in early-onset T2DM [12]. These results are consistent with our study. In the present study, there was no direct effect between the decreased TT levels and the increased age of diabetes onset, while HbA1c, BMI, and TG acted as mediating factors. The 3*β*-HSD levels also negatively associated with HbA1c. Hyperglycemia has been demonstrated as an important factor in the reduction of testosterone levels in type 2 diabetes [14, 23]. Our previous studies also demonstrated that blood glucose normalization in patients with T2DM elevated the TT levels significantly [15, 24]. Moreover, blood glucose normalization via short-term intensive insulin therapy increased 3*β*-HSD and 17*β*-HSD levels in previous study [16]. Thus, in men with newly diagnosed early-onset T2DM, higher HbA1c may lead to lower 3*β*-HSD and consequent lower TT levels compared with men with late-onset T2DM, and intensive hypoglycemic therapy may improve testosterone deficiency and prevent the following hypogonadism. On the other hand, low TT levels may aggravate the metabolic disorders and accelerate the development of diabetes [8]. Previous study showed that testosterone treatment can prevent or revert type 2 diabetes in men [25]. However, which of the two (low TT and hyperglycemia) is the priming factor of early-onset T2DM needs to be further studied. The causal relationship between sexual hormones and the age of onset of T2DM could not be concluded in the cross-sectional study, which is a limitation of this study. The problems of obesity and lipid disorder in patients with early-onset T2DM were worse than in the other patients with T2DM [12, 26] and have been demonstrated as risk factors of hypogonadism and low testosterone [14], which were also observed in the present study. The severe metabolic disorder in the patients with early-onset T2DM may lead to the unexpected age-inappropriate TT levels.

In the present study, patients with early-onset T2DM had higher fasting blood glucose than the other group, but the fasting C-peptide was similar between the two groups. This may be due to the hepatic insulin resistance or less portal levels of insulin in patients with early-onset diabetes. These patients had larger BMI and waist circumference, as well as more severe lipid disorders compared to patients with late-onset T2DM in this study, which significantly increased the likelihood of developing nonalcoholic fatty liver disease. The changes of the liver in patients with early-onset T2DM may also lead to SHBG reduction, which was found in the present study. However, the hepatic disorder in patients with early-onset T2DM needs to be further investigated.

Previous study found higher proportions of smoking and drinking in patients with early-onset T2DM compared with those in patients with late-onset T2DM [3], and tobacco smoking and alcohol drinking may increase testosterone levels in men [27, 28]. However, the proportion of smoking was slightly lower in the early-onset group, and the data of alcohol consumption was lacked in the present study. Therefore, the effects of smoking or alcohol consumption on the testosterone reduction in early-onset T2DM remain unclear, which is one of the limitation of our study.

In conclusion, newly diagnosed male patients with early-onset T2DM had lower TT levels than those with late-onset T2DM, which may be associated with the decreased level of 3*β*-HSD and more severe metabolic disorders before diabetes diagnosis in these patients. The poor glycemic control, obesity, and lipid disorder, as well as testosterone deficiency in men with early-onset T2DM, need to be treated aggressively.

## Figures and Tables

**Table 1:** General characteristics between two groups.

	Early-onset T2DM	Late-onset T2DM	*p*
Number	63	90	—
Age (year)	34.39 ± 0.66	52.41 ± 0.73	<0.001
BMI (kg/m^2^)	26.84 ± 0.50	24.94 ± 0.32	0.002
WC (cm)	94.7 ± 1.5	87.01 ± 0.85	<0.001
HbA1c (%)	10.47 ± 0.27	9.66 ± 0.22	0.022
TC (mmol/L)	5.3 (4.54, 6.02)	5.08 (4.31, 5.77)	0.116
TG (mmol/L)	2.95 (1.54, 4.25)	1.81 (1.27, 2.41)	<0.001
HDL (mmol/L)	0.93 (0.83, 1.07)	1.02 (0.92, 1.15)	0.009
LDL (mmol/L)	2.91 (2.46, 3.72)	2.87 (2.58, 3.44)	0.392
ApoA-1 (g/L)	0.96 ± 0.02	1.01 ± 0.02	0.119
ApoB (g/L)	1.11 ± 0.04	0.94 ± 0.03	<0.001
ApoA-1/ApoB	0.91 ± 0.03	1.15 ± 0.04	<0.001
FBG (mmol/L)	10.94 ± 0.5	9.75 ± 0.38	0.055
FC-p (ng/mL)	1.6 (1.06, 2.45)	1.69 (1.24, 2.1)	0.792
PC-p (ng/mL)	2.78 (1.81, 5.76)	4.06 (2.35, 5.33)	0.101
FGLA (pg/mL)	125.16 (102.14, 151.2)	125.3 (104.04, 136.86)	0.605
PGLA (pg/mL)	155.13 (132.28, 181.28)	142.34 (125.68, 169.41)	0.285
HOMA-IR	1.82 (1.14, 2.7)	1.61 (1.23, 2.15)	0.299
GAD-Ab (IU/mL)	0.76 (0.45, 1.30)	0.95 (0.43, 1.83)	0.257
ICA-Ab positive (%)	0	0	—
Smoking (%)	34.92	42.89	0.099
Hypertension (%)	25.40	33.33	0.371

BMI: body mass index; WC: waist circumference; HbA1c: glycated hemoglobin; TC: total cholesterol; TG: triglyceride; LDL: low-density lipoprotein; HDL: high-density lipoprotein; ApoA-1: apolipoprotein A-1; ApoB: apolipoprotein B; FBG: fasting blood glucose; FC-P: fasting C-peptide; PC-p: postprandial C-peptide; FGLA: fasting glucagon; PGLA: postprandial glucagon; HOMA-IR: homeostatic model assessment of insulin resistance; GAD-Ab: glutamic acid decarboxylase antibody; ICA-Ab: islet cell autoantibodies. Data were presented as mean ± SE or median (interquartile range).

## Data Availability

The data sets generated during and/or analyzed during the current study are not publicly available but are available from the corresponding authors on reasonable request.

## References

[1] Li M. Z., Su L., Liang B. Y. (2013). Trends in prevalence, awareness, treatment, and control of diabetes mellitus in mainland China from 1979 to 2012. *International Journal of Endocrinology*.

[2] Song S. H., Gray T. A. (2011). Early intensive cardiovascular risk management in young people with type 2 diabetes. *Diabetes Research and Clinical Practice*.

[3] Yeung R. O., Zhang Y., Luk A. (2014). Metabolic profiles and treatment gaps in young-onset type 2 diabetes in Asia (the JADE programme): a cross-sectional study of a prospective cohort. *The lancet Diabetes & endocrinology.*.

[4] Xu Y., Wang L., He J. (2013). Prevalence and control of diabetes in Chinese adults. *Journal of the American Medical Association*.

[5] Pavkov M. E., Bennett P. H., Knowler W. C., Krakoff J., Sievers M. L., Nelson R. G. (2006). Effect of youth-onset type 2 diabetes mellitus on incidence of end-stage renal disease and mortality in young and middle-aged Pima Indians. *Journal of the American Medical Association*.

[6] Constantino M. I., Molyneaux L., Limacher-Gisler F. (2013). Long-term complications and mortality in young-onset diabetes: type 2 diabetes is more hazardous and lethal than type 1 diabetes. *Diabetes Care*.

[7] Huo X., Gao L., Guo L. (2016). Risk of non-fatal cardiovascular diseases in early-onset versus late-onset type 2 diabetes in China: a cross-sectional study. *The lancet Diabetes & Endocrinology*.

[8] Kelly D. M., Jones T. H. (2013). Testosterone: a metabolic hormone in health and disease. *The Journal of Endocrinology*.

[9] Grossmann M. (2011). Low testosterone in men with type 2 diabetes: significance and treatment. *The Journal of Clinical Endocrinology and Metabolism*.

[10] Yassin A., Haider A., Haider K. S. (2019). Testosterone therapy in men with hypogonadism prevents progression from prediabetes to type 2 diabetes: eight-year data from a registry study. *Diabetes Care*.

[11] Feldman H. A., Longcope C., Derby C. A. (2002). Age trends in the level of serum testosterone and other hormones in middle-aged men: longitudinal results from the Massachusetts male aging study. *The Journal of Clinical Endocrinology and Metabolism*.

[12] Li Y., Zhang M., Liu X. (2017). Correlates and prevalence of hypogonadism in patients with early- and late-onset type 2 diabetes. *Andrology.*.

[13] Bhattacharya R. K., Bhattacharya S. B. (2015). Late-onset hypogonadism and testosterone replacement in older men. *Clinics in Geriatric Medicine*.

[14] Defeudis G., Mazzilli R., Gianfrilli D., Lenzi A., Isidori A. M. (2018). The CATCH checklist to investigate adult-onset hypogonadism. *Andrology*.

[15] Hu Y., Ding B., Shen Y. (2021). Rapid changes in serum testosterone in men with newly diagnosed type 2 diabetes with intensive insulin and metformin. *Diabetes Care*.

[16] Hu Y., Wang Y., Cai T. T. (2022). Short-time intensive insulin therapy upregulates 3 beta- and 17 beta-hydroxysteroid dehydrogenase levels in men with newly diagnosed T2DM. *Frontiers in Endocrinology*.

[17] Society CD (2018). Standards of medical care for type 2 diabetes in China. *Chinese Journal of Diabetes Mellitus*.

[18] Duvivier B. M., Schaper N. C., Hesselink M. K. (2017). Breaking sitting with light activities vs structured exercise: a randomised crossover study demonstrating benefits for glycaemic control and insulin sensitivity in type 2 diabetes. *Diabetologia*.

[19] Wang C., Nieschlag E., Swerdloff R. (2009). Investigation, Treatment, and Monitoring of Late-Onset Hypogonadism in Males: ISA, ISSAM, EAU, EAA, and ASA Recommendations. *European Urology*.

[20] Ganesh H. K., Vijaya Sarathi H. A., George J. (2009). Prevalence of hypogonadism in patients with type 2 diabetes mellitus in an Asian Indian study group. *Endocrine Practice*.

[21] Klinge C. M., Clark B. J., Prough R. A. (2018). Dehydroepiandrosterone research: past, current, and future. *Vitamins and Hormones*.

[22] Kaufman J. M., Vermeulen A. (2005). The decline of androgen levels in elderly men and its clinical and therapeutic implications. *Endocrine Reviews*.

[23] El-Sakka A. I., Sayed H. M., Tayeb K. A. (2008). Type 2 diabetes-associated androgen alteration in patients with erectile dysfunction. *International Journal of Andrology*.

[24] Cai T., Hu Y., Ding B. (2021). Effect of metformin on testosterone levels in male patients with type 2 diabetes mellitus treated with insulin. *Front Endocrinol*.

[25] Wittert G., Bracken K., Robledo K. P. (2021). Testosterone treatment to prevent or revert type 2 diabetes in men enrolled in a lifestyle programme (T4DM): a randomised, double-blind, placebo-controlled, 2-year, phase 3b trial. *The Lancet Diabetes and Endocrinology*.

[26] Pan J., Jia W. (2018). Early-onset diabetes: an epidemic in China. *Frontiers in Medicine*.

[27] Zhao J., Leung J. Y. Y., Lin S. L., Mary S. C. (2016). Cigarette smoking and testosterone in men and women: a systematic review and meta-analysis of observational studies. *Preventive Medicine*.

[28] Walter M., Gerhard U., Gerlach M., Weijers H. G., Boening J., Wiesbeck G. A. (2006). Controlled study on the combined effect of alcohol and tobacco smoking on testosterone in alcohol-dependent men. *Alcohol Alcohol*.

